# Supplementary material for: A Novel lncRNA Mediates the Delayed Tooth Eruption of Cleidocranial Dysplasia
Source: Cells. 2022 Sep 1;11(17):2729. doi: 10.3390/cells11172729 (PMC9454660; doi:10.3390/cells11172729)
Supplement: Supplementary file 1 [file cells-11-02729-s001.zip › cells-1886260-supplementary.pdf]

Table S1. The gene-specific primers for RACE analysis.

Primer Names	Sequences (5′-3′)
3′-RACE-F1	AGACTTCCATGTGTCGAGGAACAG
3′-RACE-F2	AGTGCCCTCTGCTGTTCATGAAGA
5′-RACE-R3	CCTGAATAGATATCACTCCGCAAC
5′-RACE-R4	TACATGAAGATAAACAGAGGGCTA

Table S2. The sequences of gRNAs.

gRNA Names	Sequences (5′-3′)
OC-lncRNA-gRNA-1	CTCCCTCACTTGGTGAAGAC
OC-lncRNA-gRNA-2	TTGCGGAGTGATATCTATTC
OC-lncRNA-gRNA-3	CCTAAGAAGCAGAGACATTC
OC-lncRNA-gRNA-4	TCCTCACCAATATAAACATT

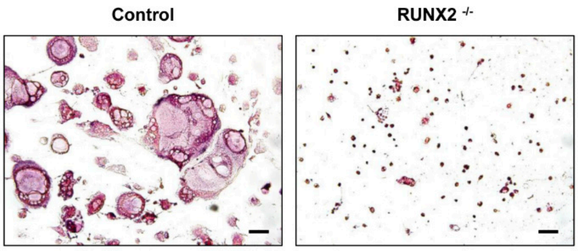

Figure. S1. TRAP staining showed *Runx2* knock-out severely impaired the osteoclast differentiation. Scale bar, 200 μm.

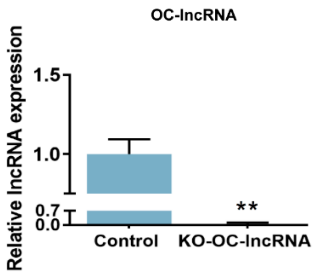

Figure. S2. Efficiency of OC-lncRNA knock-out by CRISPR/Cas9. Error bars represent SD. **P < 0.01.

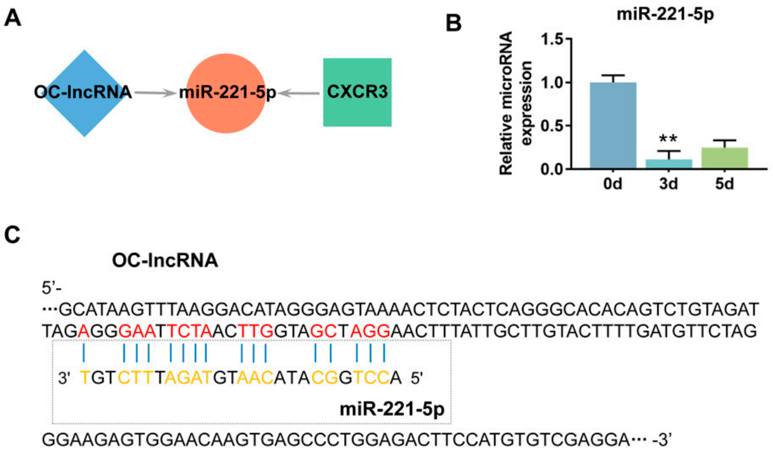

Figure S3. OC-lncRNA sponged miR-221-5p. (A) OC-lncRNA and CXCR3 competitively bound to miR-221-5p. (B) miR-221-5p expression levels in RANKL-induced osteoclast differentiation. (C) Schematic representation of the potential binding sites between OC-lncRNA and miR-221-5p. **P < 0.01.

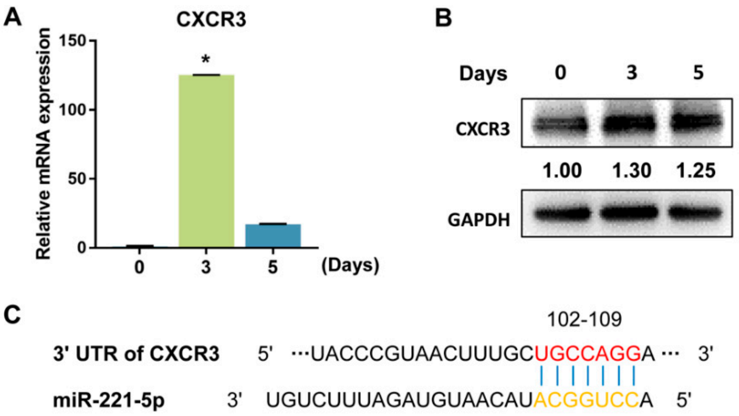

Figure S4. CXCR3 is a target gene of miR-221-5p. mRNA (A) and protein (B) expression levels of CXCR3 in osteoclast differentiation. (C) Schematic representation of the potential binding sites between OC-lncRNA and miR-221-5p. *$P < 0.05$.
